# CD133-Positive Membrane Particles in Cerebrospinal Fluid of Patients with Inflammatory and Degenerative Neurological Diseases

**DOI:** 10.3389/fncel.2017.00077

**Published:** 2017-03-27

**Authors:** Tobias Bobinger, Lisa May, Hannes Lücking, Stephan P. Kloska, Petra Burkardt, Philipp Spitzer, Juan M. Maler, Denis Corbeil, Hagen B. Huttner

**Affiliations:** ^1^Department of Neurology, University Hospital ErlangenErlangen, Germany; ^2^Department of Neuroradiology, University Hospital ErlangenErlangen, Germany; ^3^Department of Psychiatry, University Hospital ErlangenErlangen, Germany; ^4^Biotechnology Center, Technische Universität DresdenDresden, Germany

**Keywords:** CD133, cerebro-spinal fluid, multiple sclerosis, membrane particles, neurodegenerative disorders

## Abstract

**Background:** Analysis of cerebrospinal fluid (CSF) is a frequently used diagnostic tool in a variety of neurological diseases. Recent studies suggested that investigating membrane particles enriched with the stem cell marker CD133 may offer new avenues for studying neurological disease. In this study, we evaluated the amount of membrane particle-associated CD133 in human CSF in neuroinflammatory and degenerative diseases.

**Methods:** We compared the amount of membrane particle-associated CD133 in CSF samples collected from 45 patients with normal pressure hydrocephalus, parkinsonism, dementia, and cognitive impairment, chronic inflammatory diseases and 10 healthy adult individuals as controls. After ultracentrifugation of CSF, gel electrophoresis and immunoblotting using anti-CD133 monoclonal antibody 80B258 were performed. Antigen-antibody complexes were detected using chemiluminescence.

**Results:** The amount of membrane particle-associated CD133 was significantly increased in patients with normal pressure hydrocephalus (*p* < 0.001), parkinsonism (*p* = 0.011) as well as in patients with chronic inflammatory disease (*p* = 0.008). Analysis of CSF of patients with dementia and cognitive impairment revealed no significant change compared with healthy individuals. Furthermore, subgroup analysis of patients with chronic inflammatory diseases demonstrated significantly elevated levels in individuals with relapsing-remitting multiple sclerosis (*p* = 0.023) and secondary progressive multiple sclerosis (SPMS; *p* = 0.010).

**Conclusion:** Collectively, our study revealed elevated levels of membrane particle-associated CD133 in patients with normal pressure hydrocephalus, parkinsonism as well as relapsing-remitting and SPMS. Membrane glycoprotein CD133 may be of clinical value for several neurological diseases.

## Introduction

The cerebrospinal fluid (CSF) is frequently analyzed for diagnosing a variety of neurological diseases. The basic CSF-analysis includes cell count, cytological findings and total amount of protein (Johanson et al., 2011). Specific clinical CSF-analyses focus on protein-based alterations such as oligoclonal banding, particular antibodies, or particular soluble proteins such as tau (Hühmer et al., 2006). Yet, CSF may have a far greater diagnostic potential considering recent studies demonstrating that CSF contains small membrane particles (Huttner et al., 2008, 2012; Colombo et al., 2012; Chiasserini et al., 2014).

**FIGURE 1:**
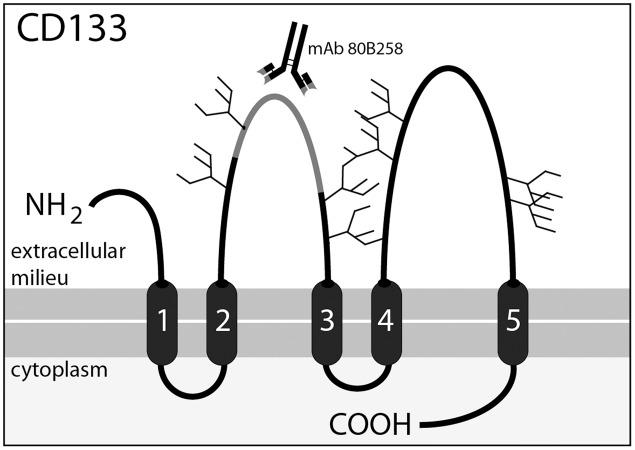
**Membrane topology of CD133 and location of the 80B258 epitope**. CD133 consists of an extracellular N-terminal domain, 5-transmembrane domains separating two large glycosylated extracellular loops and two small intracellular loops, and an intracellular C-terminal domain. Potential N-glycosylation sites are indicated with forks. Mouse-mAb 80B258 was generated against a fusion protein containing part of the first extracellular loop (residue glycine 240 to serine 388, gray segment) of human prominin-1 (Karbanová et al., 2008).

As previously shown, CSF-associated membrane particles may contain CD133, a cholesterol-binding pentaspan membrane glycoprotein (**Figure 1**). Although the biological function of this glycoprotein remains to be demonstrated, it gained attention in the past few years. CD133 labels stem and progenitor cells in various organs notably in the neural and hematopoietic system (Miraglia et al., 1997; Weigmann et al., 1997; Jászai et al., 2013; Walker et al., 2013; Pruszak, 2015). CD133 highlights cancer stem cells and marks potential facultative stem cells, which might be activated during regeneration (Grosse-Gehling et al., 2013; Corbeil et al., 2014). Besides its expression in progenitor cells, CD133 is detected in differentiated cells such as epithelial and glial cells (Karbanová et al., 2008; Corbeil et al., 2009). In the adult brain it is expressed on multiciliated ependymal cells and subventricular zone astrocytes. Therein, CD133 is concentrated in ependymal cilia as well as in primary cilia of quiescent neural stem cells that are exposed to CSF (Dubreuil et al., 2007; Coskun et al., 2008; Huttner et al., 2008; Codega et al., 2014; Karbanová et al., 2014; Nogueira et al., 2014). CD133 is also detected in myelin sheaths of oligodendrocytes and Schwann cells found in central and peripheral nervous system, respectively (Corbeil et al., 2009). A remarkable biological feature of CD133 is its release in several body fluids in association with membrane particles, initially referred to as “prominosomes” (Marzesco et al., 2005). Thus, CD133 can be recovered upon ultracentrifugation in urine, tear, saliva, seminal fluid and CSF (Marzesco et al., 2005; Huttner et al., 2008, 2012; Karbanová et al., 2008, 2014). Although the release of CD133-associated membrane particles can occur at a constant rate, particularly in healthy epithelial cells contacting a given body fluid (e.g., urine, CSF), an increase of CD133 secretion was linked to the differentiation of stem and progenitor cells (Marzesco et al., 2005; Dubreuil et al., 2007; Bauer et al., 2011). The release of CD133 in CSF can occur by the budding of membrane particles (i.e., ectosomes or microvesicles) from plasma membrane protrusions (e.g., cilia) of ependymal cells and/or underlying subventricular astrocytes (Marzesco et al., 2005; Dubreuil et al., 2007; Corbeil et al., 2009, 2013; Kriegstein and Alvarez-Buylla, 2009). The release of CD133 in association with exosomes, i.e., the internal membrane vesicles of multivesicular bodies that are discharged into the extracellular space by exocytosis (Fevrier and Raposo, 2004) cannot be excluded, particularly in cancerous cells. However, the detection of CD133 in exosomes was reported only with hematopoietic progenitors (Bauer et al., 2011).

Monitoring the amount of membrane particle-associated CD133 in body-fluids may provide information about biological activity of contacting cells and its variation in onset and progression of several diseases. To date, only two reports were published on membrane particle-associated CD133 in human CSF. In essence, these studies found an elevation of CD133 in early stage glioblastoma as well as in temporal lobe epilepsy (Huttner et al., 2008, 2012). In the light of such observations, we investigated the amount of membrane particle-associated CD133 in CSF of patients with inflammatory and degenerative neurological diseases and assessed the possible clinical value of membrane-bound CD133.

## Materials and Methods

### Study Design, Patient Selection and Retrieval of Clinical Data

The study included patients admitted to the Department of Neurology, University Hospital Erlangen (Germany) and was approved by the local institutional review board. CSF samples were collected in inpatients who received a diagnostic lumbar puncture, indicated to exclude or verify neurological disease, and consented for research-based CSF analysis.

We enrolled (i) 10 patients who received CSF analysis to exclude subarachnoid hemorrhage or inflammatory disease showing normal CSF analysis and these patients are in the following referred to as healthy subjects, (ii) patients with normal pressure hydrocephalus (*n* = 6), parkinsonism (*n* = 6), dementia and cognitive impairment (*n* = 8) and chronic inflammatory central nervous system disease (*n* = 25). Clinical data and medical information were collected from our institutional database. In patients with chronic inflammatory central nervous system disease, the Expanded Disability Status Scale (EDSS) score was assessed during the stay (Kurtzke, 1983), the disease course was classified as clinical isolated syndrome (CIS) / relapsing remitting (RR), secondary progressive (SP) or primary progressive (PP) multiple sclerosis and myelitis (Rovira et al., 2009; Montalban et al., 2010). For diagnosis of multiple sclerosis, brain images were acquired. Images were done at a 1.5-T MRI (Siemens AG, Erlangen, Germany) consisting of an axial T1- and T2-weighted spin echo sequences. Brain atrophy (Bermel and Bakshi, 2006) and lesion load measurements were scored as recently described (Ormerod et al., 1987; Petzold et al., 2016). Samples of patients with inflammatory diseases were obtained during patient’s hospitalization for relapse therapy. In patients with dementia or cognitive impairment, the total tau protein, phospho-tau and the 42 and 40 amino acid form of amyloid β were analyzed (Zetterberg, 2015).

### Isolation of CD133-Membrane Particles

Upon lumbar puncture, an aliquot (1 ml) from the collected CSF was frozen at -80°C immediately after adding protease inhibitor according to the manufacturer’s instruction (Roche Diagnostics, Cat. No. 04693116001, Mannheim, Germany). CSF samples (1 ml each) were thawed on ice and sequentially centrifuged for 30 min at 10.000 × *g* at 4°C, the resulting supernatants were collected and centrifuged for 1 h at 100.000 × *g* at 4°C. Supernatants were discarded, and 100.000 × *g* pellets were resuspended in 35 μl Laemmli buffer and analyzed by immunoblotting (see below) (Huttner et al., 2008). Extracts from human Caco-2 cells were used as positive controls and to establish the standard curve since these cells express CD133 (Corbeil et al., 2000; Marzesco et al., 2005; Huttner et al., 2008). Caco-2 cells were produced in a medium with 20% fetal calf serum, 2 mM L-glutamine, 1% non-essential amino acids, 100 U/ml penicillin, and 100 μg/ml streptomycin under a 5% CO_2_ atmosphere at 37°C. Caco-2 cells were grown until 10 days post-confluence. Cells extracts were prepared as described (Huttner et al., 2008) and the protein concentration was measured using BCA Protein Assay (BCA Protein Assay Kit Pierce, Thermo Fisher, Cat. No. 23252, Waltham, MA, USA). The Caco-2 cell extract used had a protein concentration of 10 μg/μl and was diluted 1:100.

### Quantitative Immunoblotting

Probes and positive controls were subjected to Tris-Glycine gels (Precise 8% Tris-Glycine Gels, ThermoFisher, Cat. No. 25260, Waltham, MA, USA) and transferred to polyvinylidene difluoride membranes (Immobilon-P membrane, Merck Millipore, IPVH00010, 0.45 μm, Billerica, MA, USA) as published (Huttner et al., 2008, 2012). After blocking for 1 h in PBS containing 0.3% Tween-20 and 5% low-fat milk powder (blocking solution), membranes were incubated for 12 h in blocking solution containing mouse monoclonal antibody (mAb) 80B258 (1 μg/ml; isotype IgG1) (Karbanová et al., 2008). Membranes were then washed thrice for 15 min in PBS containing 0.3% Tween-20 and incubated for 1 h in blocking solution containing horseradish peroxidase conjugated goat anti-mouse secondary antibody (1:5000; Jackson Immunoresearch Laboratories, Cat. No. 115-035-068, West Grove, PA, USA). The antigen-antibody complexes were detected using Chemiluminescence reagents (SuperSignal West Femto Substrate, ThermoFisher Scientific, Cat. No. 34095, Waltham, Massachusetts, USA). Total transferred proteins were detected using Ponceau staining (Ponceau S solution, Sigma-Aldrich, Cat. No. P7170, St. Louis, MO, USA) (Supplementary Figures 1, 2). For visualization of antigen-antibody complexes, the image reader Fusion FX7 Spectra (Vilber Lourmat GmbH, Eberhardzell, Germany) was utilized, and quantification was performed using Image Quant TL (GE Healthcare Europe GmbH, Freiburg, Germany). Absorbance values within the linear range were used for statistical analysis as described (Huttner et al., 2012). An internal standard sample was established using the standard curve derived from Caco-2 cell extract and evaluated on each blot (Supplementary Figure 2). The absorbance levels of analyzed CSF samples of diseased individuals were compared to the mean absorbance level of healthy individuals and expressed as a deviation in percentages (CD133-deviation).

### Statistical Analysis

Data were processed using Office 2013 software package (Microsoft Corp., Redmond, WA, USA). Statistical analysis was performed with SPSS statistical software package 21.0 (SPSS Inc., Chicago, IL, USA). Distribution of the data was established using the Kolmogorov–Smirnov test. Normal distributed data are presented as mean ± standard deviation (SD) as well as standard error of the mean (SEM) (compared using Student’s *t*-test). Other data are shown as median and interquartile ranges (compared using Mann–Whitney *U*-test). Absorbance values of each group are calculated referring to healthy subjects. A logistic regression model was used to investigate the relationship between CD133-deviation and basic CSF analysis as well as EDSS-score and lesion load. All statistical tests were two-sided with a significance level of α = 0.05 without adjustment for multiple comparisons.

## Results

### Detection of CD133-Positive Membrane Particles in the CSF of Healthy and Diseased Patients

The amount of membrane particle-associated CD133 in the CSF of healthy subjects was determined upon differential centrifugation of CSF followed by semi-quantitative immunoblotting of the resulting fractions. The CD133 glycoprotein was detected using our characterized mouse mAb 80B258, which recognized a polypeptide epitope located in its extracellular loop (**Figure 1**) (Karbanová et al., 2008). The blotting was performed by analyzing three defined amounts of a CD133-containing Caco-2-cell extract (for technical details and the establishment of standard curve see Materials and Methods, Supplementary Figures 1, 2). Such controls were used to establish an internal reference. From 1 ml aliquot of CSF, we recovered 1.319 × 10^3^ ± 682 × 10^3^ pixel/ absorbance units of CD133 immunoreactivity in healthy subjects. This amount was used as a reference to healthy subjects to determine the variation of CD133 in CSF of diseased patients.

**FIGURE 2:**
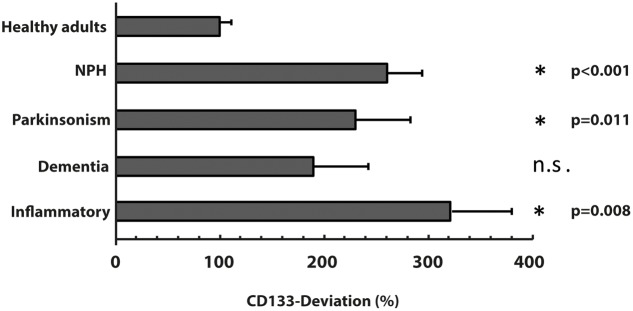
**Variation of membrane particle-associated CD133 in neurological diseases**. Variations of percentage of CD133-deviation in patients with normal pressure hydrocephalus (NPH), parkinsonism, dementia and inflammatory diseases by comparison to healthy adults are depicted in bar graph. Data are mean ± SEM. N.s.: not significant.

**FIGURE 3:**
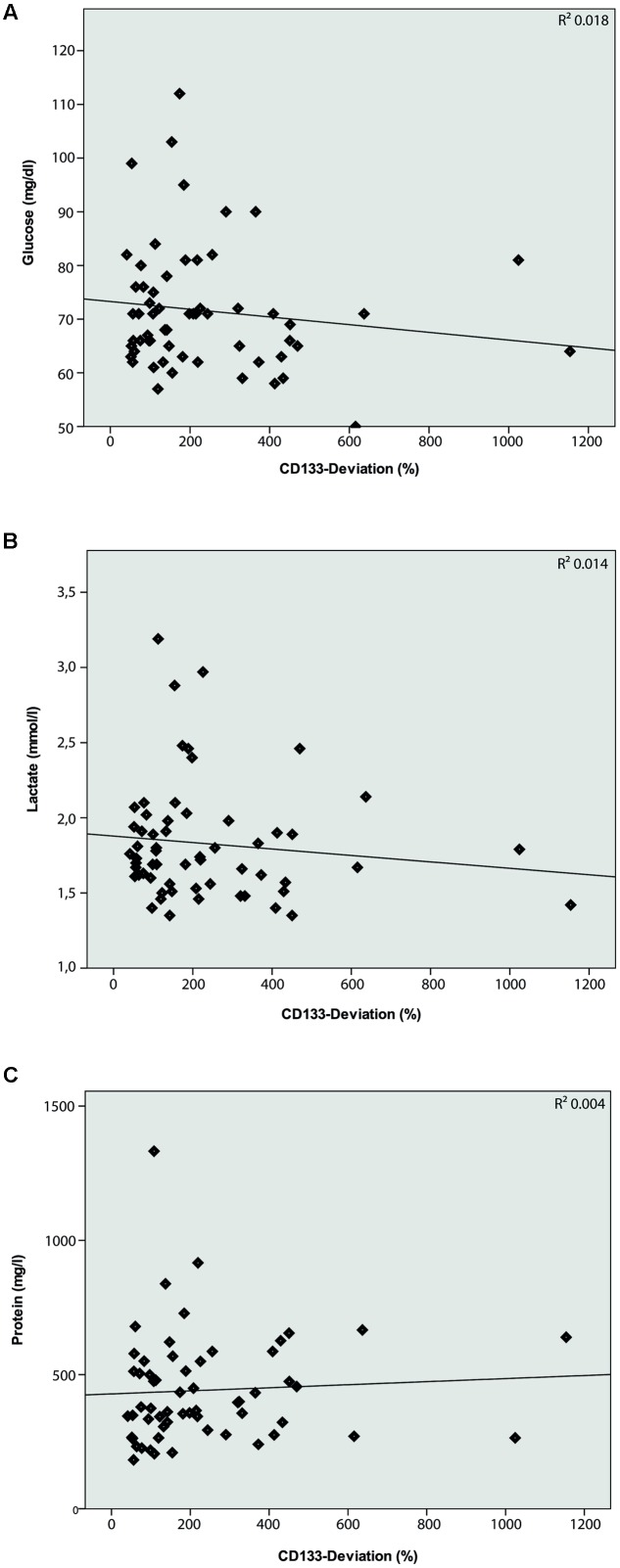
**Logistic regression model between CD133-deviation (%) and basic CSF-analysis**. Logistic regression model comparing CD133-deviation (%) and Glucose (**A**; *R*^2^ = 0.018; *p* = 0.312), Lactate (**B**; *R*^2^ = 0.014; *p* = 0.365), and Protein (**C**; *R*^2^ = 0.004; *p* = 0.643). No correlation is found between these parameters.

The characteristics of all included patients with normal pressure hydrocephalus, Parkinson’s disease, dementia and cognitive impairment, and chronic inflammatory disease are summarized in **Tables 1**, **2**. Basic parameters of CSF-analysis is provided in the Supplementary Table 1. Interestingly, there was a significant increase of CD133 in CSF collected from six patients with normal pressure hydrocephalus [261% ± 81.5% (*SD*) or 42% (SEM); *p* < 0.001] and six others with parkinsonism [230% ± 69% (*SD*) or 53%; *p* = 0.011]. In the latter cases, the increase of CD133 was observed in patients with atypical parkinsonism [two patients; 357% ± 90% (*SD*) or 54% (SEM); *p* = 0.03], patients with parkinson disease showed only a trend toward increased CD133 [four patients; 167% ± 60% (*SD*) or 30% (SEM); *p* = 0.076]. CSF-associated CD133 levels were also elevated in eight patients with dementia/cognitive impairment [190% ± 150% (*SD*) or 52% (SEM)] but the increase is not significant by comparison to healthy subjects (*p* = 0.237). Lastly, the analysis of 25 patients with chronic inflammatory diseases revealed an elevated amount of CD133 in their CSF [321% ± 210% (*SD*) or 58% (SEM); *p* = 0.008] (**Figure 2**). Regression analysis revealed no association between CD133-deviation and parameters of basic CSF-analysis (glucose, lactate, protein) (**Figure 3**).

**Table 1:** Clinical parameters of healthy subjects, patients with normal pressure hydrocephalus, parkinsonism, and dementia/cognitive impairment.

Parameters	Values
*Healthy subjects (n = 10)*	
Age (years), median (range)	53 (49.3–61.5)
Sex (female/male)	6/4
Reason for lumbar puncture (*n*)	
Exclusion of subarachnoid hemorrhage	2 (20%)
Exclusion of inflammatory disease	8 (80%)
*Normal pressure hydrocephalus (n = 6)*	
Age (years), median (range)	72 (66–78)
Sex (female/male)	1/5
*Parkinson syndrome (n = 6)*	
Age (years), median (range)	73.0 (68.5–78.5)
Parkinson disease (%)	4 (66.67%)
Atypical parkinsonism (%)	2 (33.34%)
*Dementia / cognitive impairment (n = 8)*	
Age (years), median (range)	70.4 (68.5–75.0)
Sex (female/male)	4/4
ß-Amyloid 1–42 pg/ml (range)	821 (687–1232)
Ratio ß-Amyloid 1–42/1–40	0.037 (0.031–0.251)
Total tau pg/ml (range)	486.5 (358.0–749.5)
Phosphor tau pg/ml (range)	76.3 (48.6–94.0)

**Table 2:** Clinical parameters of the included patients with chronic inflammatory disease.

Parameters	Values
*Subjects with central nervous inflammatory disease (n = 25)*	
Age (years), median (range)	47.5 (41.25–53.75)
Sex (female/male)	(17/8)
CIS / RRMS *n* (%)	7 (28.0%)
SPMS *n* (%)	10 (40.0%)
PPMS *n* (%)	5 (20.0%)
Myelitis *n* (%)	3 (12.0%)
Current EDSS score median (range)	5.0 (2.0–6.5)
Disease duration (months), median (range)	120 (27–249)
*Neuroradiological data (n = 25)*	
*T2 lesions*	
Periventricular	
Lesion 6–10 mm (%)	62.5% (15)
Lesion >10 mm (%)	50.0% (12)
Juxtacortical	
Lesion 6–10 mm (%)	66.7% (16)
Lesion >10 mm (%)	25.0% (6)
Infratentorial	
Lesion 6–10 mm (%)	29.1% (7)
Lesion >10 mm (%)	16.7% (4)
Score (range)	13.3 (8–21)
Spinal T2 lesions (n = 17)	76.5% (13)
T1 CE lesions	
Cerebral lesions	16.7% (4)
Spinal lesions	8.3% (2)
Cerebral atrophy	
Bicaudate ratio	0.12 (0.098–0.135)

### Subgroups of Chronic Inflammatory Disease

Given that patients with chronic inflammatory diseases showed the highest increase in CSF-associated CD133 (**Figure 2**), we performed a sub-analysis and grouped these 25 patients into relapsing-remitting multiple sclerosis (RRMS, including CIS; *n* = 7), secondary progressive multiple sclerosis (SPMS, *n* = 10), primary progressive multiple sclerosis (PPMS, *n* = 5) and patients with isolated myelitis (*n* = 3). Baseline characteristics, clinical and radiological parameters are shown in **Table 2**.

**FIGURE 4:**
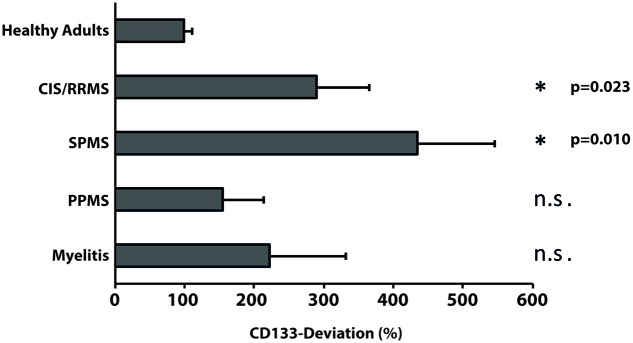
**Variation of membrane particle-associated CD133 in neuroimmunological diseases**. Variations of percentage of CD133-deviation in patients with clinical isolated syndrome/relapsing remitting multiple sclerosis (CIS/RRMS), secondary progressive multiple sclerosis (SPMS), primary progressive multiple sclerosis (PPMS) and myelitis patients by comparison to healthy adults are depicted in bar graph. Data are mean ± SEM. N.s.: not significant.

**FIGURE 5:**
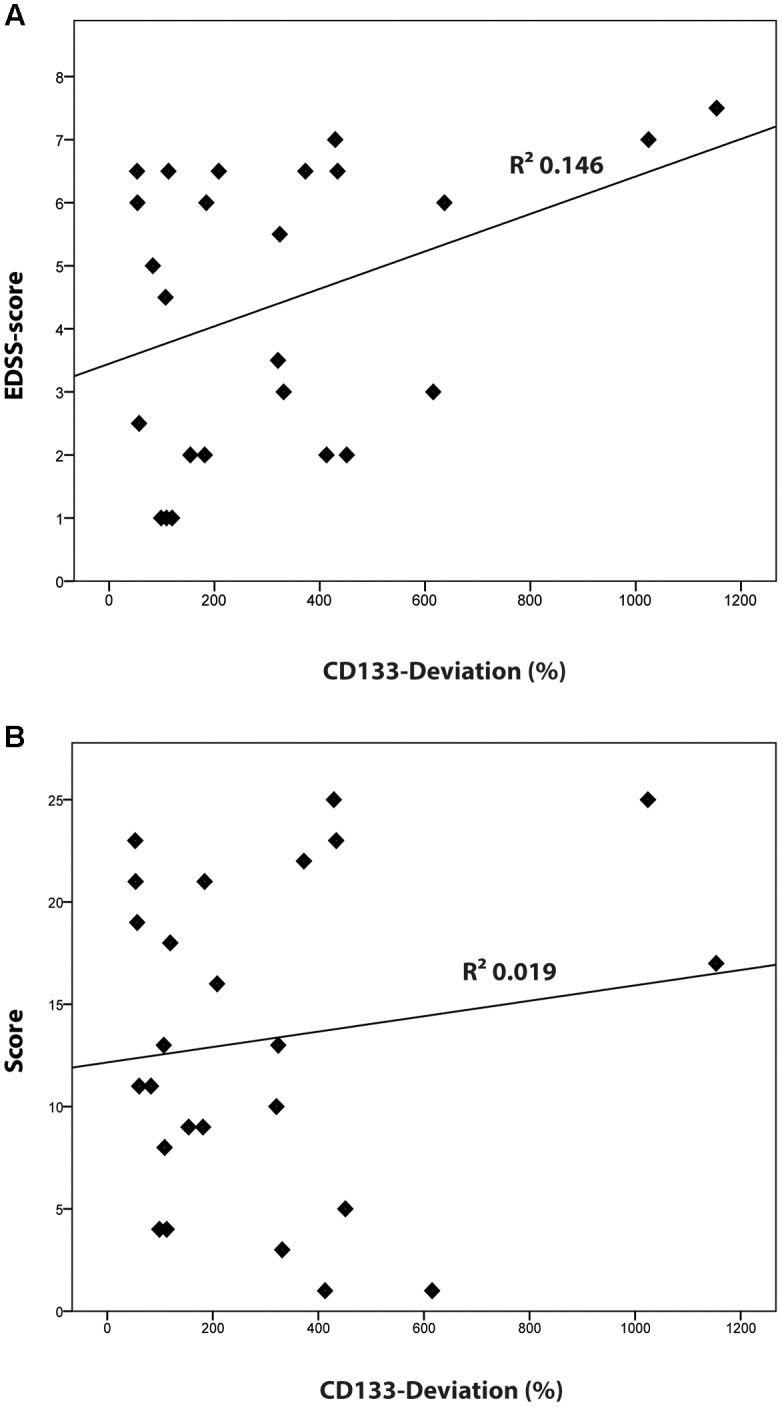
**Correlation of CD133 with clinical [Expanded Disability Status Scale (EDSS)-score] and radiological findings (lesion score). (A)** Logistic regression model comparing the percentage of CD133-deviation and EDSS score (*R*^2^ = 0.146; *p* = 0.059). **(B)** Logistic regression model comparing the percentage of CD133-deviation and the radiological lesion score (*R*^2^ = 0.019; *p* = 0.513).

Compared to healthy controls, levels of CD133 immunoreactivity in CSF revealed no increase in PPMS and myelitis-patients [155% ± 90% (*SD*) or 59% (SEM); *p* = 0.768; and 222% ± 134% (*SD*) or 109% (SEM); *p* = 0.273, see **Figure 4**]. In contrast, patients with RRMS and SPMS showed increased CD133-levels compared to healthy controls [291% ± 91% (*SD*) or 76% (SEM); *p* = 0.023], and 434% ± 135% (*SD*) or 111% (SEM); *p* = 0.010, respectively. Finally, further analysis revealed no correlation between radiological parameters (lesion load / T2 lesions / T1 CE lesions / cerebral atrophy) [*R*^2^ = 0.019, *p* = 0.513] and CD133-deviation, however, a statistical trend was seen when comparing EDSS-score with CD133 (*R*^2^ = 0.146, *p* = 0.059, see **Figure 5**).

## Discussion

The present study for the first time systematically investigated human CSF in inflammatory and degenerative diseases for membrane particles carrying CD133. In essence, patients with parkinsonism, normal pressure hydrocephalus as well as with relapsing-remitting multiple sclerosis and SP multiple sclerosis showed markedly increased CD133 levels. Several aspects emerge from the data.

On a general note, analysis of membrane particles within the human CSF – compared to solely protein-based CSF investigation – may offer new approaches to study CNS diseases. CD133-positive membrane particles occur in CSF most likely as ectosomes, i.e., membrane particles derived from plasmalemma notably membrane protrusions. Specific CNS cells, such as ependymal cells and subventricular zone astrocytes which extend their cilium into the lumen, represent a plausible source of these CD133-positive membrane particles (Marzesco et al., 2005; Dubreuil et al., 2007; Corbeil et al., 2009, 2013; Kriegstein and Alvarez-Buylla, 2009). At the current stage of investigation, we cannot rule out the contribution of infiltration cells in the CSF-associated CD133 production. In this regard it has to be kept in mind that our study did not reveal a correlation between basic CSF parameters like glucose, lactate, protein and CD133.

CD133 is also expressed by endothelial progenitor cells (Peichev et al., 2000). The diseases analyzed in this study may display processes of neovasculogenesis (Muramatsu et al., 2012) and/or blood-brain barrier dysfunction (Stolp and Dziegielewska, 2009). The presence of CD133 in myelin should not be neglected as well. Damage or remodeling of myelin sheaths might also contribute to the release of CD133-positive membrane particles by glia cells. In several diseases, a contribution of various cellular types and processes might account for the elevation of CD133 in CSF.

Given the fact that CD133 is associated to these CSF membrane particles, it has important clinical implications of a membrane particle-based CSF analysis for several reasons. Astrocytes and especially their reduced astrocytic response have been linked to neurodegenerative disease such as Alzheimer’s disease and amyotrophic lateral sclerosis (Li et al., 2016). Results suggest that astrocytes affect oligodendrocytes via Aß pathways in Alzheimer’s disease, moreover, they are linked to progressive loss of corticospinal and spinal motor neurons by progressive reactive astrogliosis and reduction in myelin (Kang et al., 2013; Fu et al., 2015; Li et al., 2016). As shown here, also Parkinson’s disease patients show increased CSF-associated CD133. Though, it remains speculative, if degeneration of myelinated axons from the substantia nigra may contribute to elevated levels of myelin-derived CD133. As patients in the different groups differ by age, this raises the question of an additional influence of age on membrane particle associated CD133. However, our previous study demonstrated, that the concentration of CD133 is constant over age and this fact could not account for elevated levels of CD133 in several groups (Huttner et al., 2008).

Specifically interesting is the finding of increased CD133 in multiple sclerosis, as CD133 was found in myelin sheaths of glial cells (Corbeil et al., 2009). In multiple sclerosis, proinflammatory cytokines are assumed to attack pathogenic cells and destroy oligodendrocytes, myelin, and axons. Then protective trophic factors may modify the blood-brain barrier and modulate the extracellular matrix (Nait-Oumesmar et al., 2008; Guo et al., 2011; Kummerfeld et al., 2012; Cristofanilli et al., 2014; Li et al., 2016; Ludwin et al., 2016). Reactive gliogenesis on the one hand, massive myelin destruction with the release of myelin-derived CD133 into CSF on the other hand, may explain the high CD133-levels in multiple sclerosis, especially in those patients with a very high disease activity. It remains to be determined whether the inflammation process itself stimulates the release of CD133 and/or activates its expression. Interestingly, our analysis demonstrated a statistical trend for an elevation of CD133 in higher EDSS-scores. A relation between the inflammation and CD133 expression was noted in solid cancers (Karbanová et al., 2014; Rappa et al., 2015). Altogether, CD133 may serve as a surrogate marker for monitoring disease activity – similar to radiological findings – after initiation of immunomodulatory therapy (Harris and Sadiq, 2014; Khan et al., 2016; Uher et al., 2016; Zivadinov et al., 2016).

There are some shortcomings of the present study, notably the lack of repeated CSF analyses for longitudinal CD133 assessment. Moreover, findings would be more robust if they are replicated in a larger cohort of independent patients. Yet, results may have been biased by analysis of semi-quantitative immunoblotting, instead of ELISA or stronger methodological approaches, leaving room for some technical uncertainty and reliability. Finally, to determine the exact cellular source(s) of the membrane particles and their origin (plasma membrane versus multivesicular bodies), we would need an exhaustive characterization (e.g., proteomics and/or lipidomics) of CD133-positive membrane particles isolated from each patient group (Redzic et al., 2014). These additional lines of investigation can extend our observation to other biomarkers, e.g., non-coding RNAs, and reveal discrete differences in the composition of membrane particles in various neurological disease (Tietje et al., 2014). So far, it remains unclear, if the isolated membrane particles are solely ectosomes or mixture of them with exosomes. Labeling for exosomal markers in future studies may solve this issue. Taken together, our results not only indicate a possible clinical value (e.g., biomarker for monitoring of several diseases), but also emphasize the need for further studies addressing all these shortcomings.

## Conclusion

Our study revealed elevated levels of membrane particle-associated CD133 in patients with normal pressure hydrocephalus, parkinsonism, relapsing-remitting and secondary-progressive multiple sclerosis. Thus, CD133 may be of clinical value for several neurological diseases.

## Availability of Data and Material

All data supporting this study are included in this published article and its supplementary information files.

## Ethics Statement

The study was approved by the local institutional review board and informed consent was obtained from the participant.

## Author Contributions

TB: study concept, data acquisition, analysis and interpretation of data, writing the manuscript. LM: data acquisition, analysis and interpretation of data. HL: data acquisition, analysis and interpretation of data, critical revision of manuscript. SK: data acquisition, analysis and interpretation of data, critical revision of manuscript. PB: data acquisition, interpretation of data, critical revision of manuscript. PS: data acquisition, interpretation of data, critical revision of manuscript. JM: data acquisition, interpretation of data, critical revision of manuscript. DC: data acquisition, interpretation of data, critical revision of manuscript. HH: study concept, data acquisition, analysis and interpretation of data, writing and revision of manuscript.

## Conflict of Interest Statement

The authors declare that the research was conducted in the absence of any commercial or financial relationships that could be construed as a potential conflict of interest.
