# Supplementary material for: CD133-Positive Membrane Particles in Cerebrospinal Fluid of Patients with Inflammatory and Degenerative Neurological Diseases
Source: Front Cell Neurosci. 2017 Mar 27;11:77. doi: 10.3389/fncel.2017.00077 (PMC5366322; doi:10.3389/fncel.2017.00077)
Supplement: Supplementary file 1 [file Table_1.PDF]

Supplementary Material - Table

CD133-positive membrane particles in cerebrospinal fluid of patients with inflammatory and degenerative neurological diseases

Tobias Bobinger*, Lisa May, Hannes Lücking, Stephan P. Kloska, Petra Burkardt,

Philipp Spitzer, Juan M. Maler, Denis Corbeil, Hagen B. Huttner

*** Correspondence:** Tobias Bobinger: Tobias.Bobinger@uk-erlangen.de

1 Supplementary Table 1 – CSF parameters of all patients

**Parameters**	**Values**
Healthy subjects (n=10)	
Cell count	1.0 (1.0-2.3)
Glucose	67.5 (62.8-75.8)
Protein	328.5 (256.0-365.5.0)
Lactate	1.6 (1.4-1.9)
Normal pressure hydrocephalus (n=6)	
Cell count	3.0 (0.8-5.0)
Glucose	76.5 (71.0-84.0)
Protein	531.0 (336.8-586.0)
Lactate	2.2 (1.7-2.6)
Parkinson syndrome (n=6)	
Cell count	1.5 (1.0-2.3)
Glucose	67.0 (64.3-71.0)
Protein	477.0 (347.8-857.5)
Lactate	1.6 (1.4-2.1)
Dementia / cognitive impairment (n=8)	
Cell count	3.0 (2.0-3.8)
Glucose	78.5 (66.0-88.0)
Protein	433.0 (344.3-610.3)
Lactate	1.7 (1.5-1.8)
Chronic inflammatory disease (n=25)	
Cell count	4 (1-10)
Glucose	67.0 (61.8-73.8)
Protein	376.0 (264.0-521.5)
Lactate	1.7 (1.5-1.9)
