# Supplementary material for: CD133-Positive Membrane Particles in Cerebrospinal Fluid of Patients with Inflammatory and Degenerative Neurological Diseases
Source: Front Cell Neurosci. 2017 Mar 27;11:77. doi: 10.3389/fncel.2017.00077 (PMC5366322; doi:10.3389/fncel.2017.00077)
Supplement: Supplementary file 2 [file Image_1.PDF]

Supplementary Material - Figures

CD133-positive membrane particles in cerebrospinal fluid of patients with inflammatory and degenerative neurological diseases

Tobias Bobinger*, Lisa May, Hannes Lücking, Stephan P. Kloska, Petra Burkardt,

Philipp Spitzer, Juan M. Maler, Denis Corbeil, Hagen B. Huttner

*** Correspondence:** Tobias Bobinger: Tobias.Bobinger@uk-erlangen.de

Supplementary Figures 1-2

1 Supplementary Figure 1

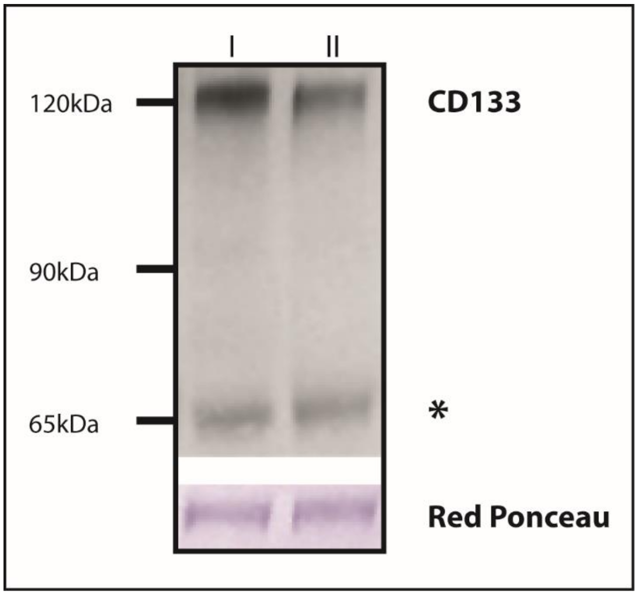

Supplementary Figure 1: Immunodetection of CSF-associated CD133 upon ultracentriguation

Pellets of CSF (1ml) recovered after 1 hour centrifugation at 100.000g were subjected to immunoblotting for CD133 (120kDa) using mAb 80B258 (top panel) and Red Ponceau staining (bottom panel). CSF from two distinct individuals (I and II) were processed. Asterisks indicate membrane-absorbed albumin (65 kDa). Note the variation of CD133 immunoreactivity in two patients while similar amounts of albumin and Red Ponceau-labeled protein were observed. Molecular mass markers (kDa) are indicated.

2 Supplementary Figure 2

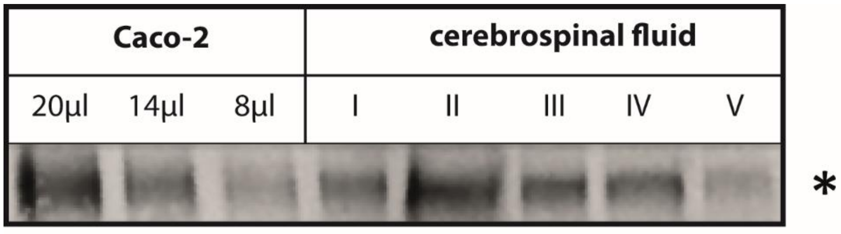

Supplementary Figure 2: Standard curve of CD133 immunoreactivity

Three different amounts (20, 14, and 8 µl; 100 ng of protein per µl) of a Caco-2 membrane extract were subjected to immunoblotting for CD133 using mAb 80B258 and the corresponding construct a standard curve. For example, CSF samples from five (I-V) individuals were analyzed.
